# RE: Preoperative serum albumin as a prognostic factor in patients with upper urinary tract urothelial carcinoma

**DOI:** 10.1590/S1677-5538.IBJU.2015.0102

**Published:** 2015

**Authors:** Ja Hyeon Ku, Myong Kim, Woo Suk Choi, Cheol Kwak, Hyeon Hoe Kim

**Affiliations:** Department of Urology, Seoul National University College of Medicine, Seoul, Korea

*To the editor*,

The publication on “Preoperative serum albumin and upper urinary tract urothelial carcinoma” is very interesting (1). Ku et al. reported that “hypoalbuminemia is an independent marker of poor prognosis in patients with upper urinary tract urothelial carcinoma (1).” Indeed, there are many recent reports confirming the importance of pretherapeutic serum albumin level (1, 2). This parameter can represent that nutritional status of the patient which is an important basic determinant for ability of the body to fight with illness (2). Nevertheless, there are many factors that can affect the serum albumin level.

The good examples are the laboratory interferences and artifacts (such as fibrinogen and heparin) that can alter the serum albumin result (3, 4). In addition, to assess the nutritional status preoperatively, it is suggested not only serum albumin but also other parameters such as hemoglobin has to be concurrently determined and interpreted (5). As noted by Delmore, “the Prognostic Nutritional Index (PNI), including serum parameters, immune competence testing and anthropometrics (6)” is recommended for “predicting postoperative morbidity/mortality and of selecting cancer patients (6)” Finally, there are also other confounding factors that can alter the final outcome results such as the superimposed nosocomial infection during admission, concurrent anemia, etc.

*Viroj Wiwanitit, MD, PhD* *Surin Rajabhat University, Surin, Thailand* *E-mail: wviroj@yahoo.com*
